# Association Between Serum Insulin and C-Peptide Levels and Breast Cancer: An Updated Systematic Review and Meta-Analysis

**DOI:** 10.3389/fonc.2020.553332

**Published:** 2020-10-29

**Authors:** Manwen Li, Limin Song, Junhua Yuan, Di Zhang, Caishun Zhang, Yuan Liu, Qian Lin, Haidan Wang, Kaizhen Su, Yanrun Li, Zhengye Ma, Defeng Liu, Jing Dong

**Affiliations:** ^1^ Special Medicine Department, School of Basic Medicine, Qingdao University, Qingdao, China; ^2^ Clinical Medicine Department, Medical College, Qingdao University, Qingdao, China; ^3^ Physiology Department, Medical College, Qingdao University, Qingdao, China

**Keywords:** breast cancer, C-peptide, insulin, review, meta-analysis

## Abstract

**Background:**

Several studies have reported that hyperinsulinemia plays a part in the etiology of breast cancer. However, no consensus has been reached. Therefore, we conducted a meta-analysis to explore the role of insulin and C-peptide in breast cancer.

**Methods:**

A systematic search in PubMed, Embase, and The Cochrane Library was conducted up to September, 2020. Standardized mean differences (SMDs) with 95% confidence intervals (CIs) were used to measure effect sizes. Publication bias was assessed using the Egger test. Stability of these results was evaluated using sensitivity analyses.

**Results:**

Fourteen articles including 27,084 cases and five articles including 2,513 cases were extracted for serum insulin levels and C-peptide levels. We found that C-peptide levels were positively associated with breast cancer with overall SMD = 0.37 (95% CI = 0.09–0.65, I^2^ = 89.1%). Subgroup analysis by control source illustrated a positive relationship between breast cancer and C-peptide levels in population-based control. Subgroup analysis by C-peptide level indicated a positive correlation between breast cancer and C-peptide levels no matter C-peptide levels in case group is ≤3 ng/ml or >3 ng/ml. Subgroup analysis by age showed that C-peptide level positively correlated to breast cancer in women between the ages of 50 and 60. However, we did not identify any relationship between breast cancer and insulin levels (SMD = 0.22, 95% CI = −0.06–0.50, I^2^ = 97.3%).

**Conclusion:**

This meta-analysis demonstrated that C-peptide levels were positively related to breast cancer in women, and no relationship between insulin levels and breast cancer was found.

## Introduction

There is a high mortality of breast cancer in women aged 20 to 50 years, with an incidence rate up to 10.4% of all cancers (1). The yearly incidence of the disease is increasing while the related mortality is steadily decreasing (2). Many aspects, including genetic, environmental, and hormonal factors, were involved in breast cancer. Obesity, diabetes, and other metabolic disorders have long been known to have a relationship with increased breast cancer incidence (3). Currently, the relationship between causative factor of metabolic diseases and breast cancer has been extensively studied. Metabolic hormones such as c-peptide and insulin are believed to play a role in the occurrence and development of breast cancer. The findings, however, have been inconsistent (4).

Insulin is a kind of protein growth factor secreted by the pancreas, which has physiological functions such as regulating cellular metabolism, glucose consumption, and cardiovascular homeostasis (5). There are several lines of evidence to suggest that insulin levels are positively related to breast cancer incidence. Insulin is a potent hormone that activates various pathways to drive the occurrence and progression of breast cancer (6). Obesity-induced insulin resistance is a risk factor for aggressive breast cancer biology in post-menopausal women (7). High serum insulin levels can drive breast cancer incidence, and this explains the association between obesity and breast cancer (8). However, Cordero-Franco et al. (9) reported an absence of a link between IR and breast cancer incidence, which is similar to the results presented by Mink et al. (10) and Muti et al. (11) who found no relationship between increased breast cancer risk and insulin.

C-peptide is a small peptide that connects two proinsulin molecule chains and is dissociated before insulin is released. It is secreted in equal molar quantities from pancreatic beta-cells in the insulin cycle (12). C-peptide is a biological by-product of insulin processing; however, due to its stability in plasma, c-peptide is also clinically used to evaluation of beta-cell function (13). Recent work highlighted the role of C-peptide in diabetes and also in many related complications such as cancer (14). Pisani in 2008 reported that the 26% elevated breast cancer incidence was linked to the elevated insulin or C-peptide blood levels (15), while Autier (16) reported no association between C-peptide levels and breast cancer incidence.

Because many studies link obesity, insulin resistance to breast cancer, we need to clarify the status of insulin or c-peptide in breast cancer biology. To do so, we conducted a meta-analysis and investigated whether they play a crucial role in breast cancer biology.

## Methods

### Search Strategy

Databases including PubMed, Cochrane Library, and Embase were searched by authors ML and LS independently until September, 2020. Three keywords, including medical subject heading terms and free-text terms, were used in the search strategy to find the related studies. In addition, the list of references to relevant studies was manually checked to add additional studies. Meta-analysis of Observational Studies in Epidemiology (MOOSE) guidelines were followed in the present meta-analysis.

### Study Selection and Criteria

The inclusion criteria were as follows: (1) case–control study, nested case–control study or cohort study; (2) new diagnoses with breast cancer pathologically prior to receiving any treatment; (3) adequate data to calculate standardized mean difference (SMD) with 95% confidence interval (CI); and (4) blood samples were collected after fasting overnight and before any therapeutic intervention. The exclusion criteria were as follows: (1) reviews, comments, protocols, meeting abstracts, case report, or letters; (2) no control subjects or other essential information; (3) patients in the studies had previous malignancies or co-existing diseases such as obesity and diabetes; and (4) articles not published in English.

### Data Extraction and Quality Assessment

Three researchers extracted useful information and data and evaluated the availability of individual studies independently.

Items were collected as follows: first author’s name, time of publication, countries, research type, serum insulin or C-peptide levels, measuring method, sample size, patient age, body mass index (BMI), parity, source of cases in the control group in the study (control source), and menstrual status. All discrepancies were resolved by consensus.

We used the Newcastle–Ottawa Scale (NOS) to judge the availability of all studies (17). We graded each article with a range from zero (the lowest) to nine (the highest), and if the score is ≥7, it is considered high-quality research.

### Statistical Analysis

Data from the included studies were combined for meta-analysis using Stata 16.0 (StataCorp LLC, College Station, TX, USA). The SMDs and 95% CI of each study were calculated according to the sample size, and the mean and standard deviation (SD) of insulin or c-peptide levels. The I^2^ statistic was used to investigate heterogeneity among studies (18), and a random-effect model was selected. To identify the source of heterogeneity, subgroup analysis was conducted according to control source, C-peptide level, country, measuring method, menopausal status, and age. Sensitivity analysis was also conducted to evaluate the stability of the analysis. Egger’s test was used to evaluate publication bias.

## Results

### Literature Search

**Figure 1:**
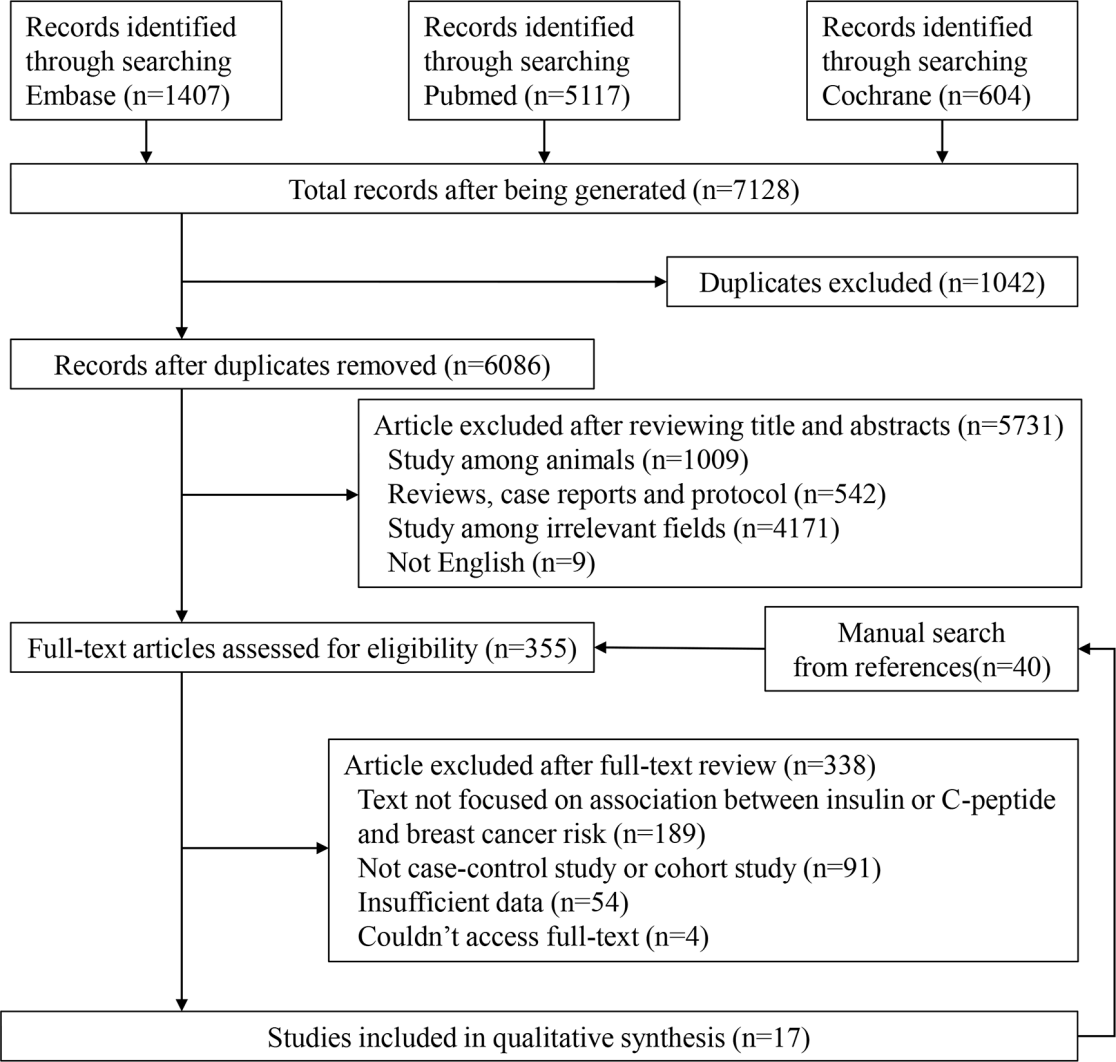
Flow diagram of the study selection process.

We presented detailed steps for the literature search in **Figure 1**. The original literature retrieval was 7,128; however, 1,042 were eliminated because of duplication. After independently selecting titles and abstracts, the two reviewers excluded 5,731 articles and 355 articles were evaluated for eligibility. Ultimately, 17 studies were included (9, 11, 19–33). We found a total 40 articles from references of relevant studies and showed them in the flow chart, but they were all included in the retrieved articles (**Figure 1**).

### Study Characteristics

A total of five articles reporting c-peptide and breast cancer were enrolled in the meta-analysis. These articles were published from 1999 to 2008 and included 2,513 women in total, of which 864 had newly diagnosed breast cancer patients in the case groups and 1,649 were people without breast cancer in control group (four case–control studies and one cohort study). There are 14 articles published in 1998 to 2018 including 27,084 women reporting information of insulin and breast cancer, of which 3,232 were newly diagnosed breast cancer patients in the case group, and 23,852 were those without breast cancer in the control group (ten case–control study, two nested case–control study, and two cohort study).

Two articles were used by both meta-analyses. Serum c-peptide and insulin in all studies were measured after overnight fasting by enzyme linked immunosorbent assay (ELISA), Radioimmunoassay (RIA), and chemiluminescence enzyme immunoassay (CLEIA). The three authors independently reviewed and cross-checked the articles, and all agreed that the research was qualified. The information was presented in **Tables 1** and **2**.

**Table 1:** Characteristics of studies included in the meta-analysis.

Author	Year	Country	Quality score	Research type	Recruiting year	Sample size		Age (mean ± SD)		BMI (mean ± SD)		Parity (mean ± SD)		Source of control*
						Cases	Control	Cases	Control	Cases	Control	Cases	Control	
C-peptide														
Helena Jernström et al.	1999	USA	8	Case control	1972–1994	45	393	74.02 ± 8.29	74.32 ± 9.63	25.14 ± 3.40	24.65 ± 4.47	1.73 ± 1.32	1.91 ± 1.58	Population
Paolo Toniolo et al.	2000	USA	8	Cohort	1985–1995	287	706	50.85 ± 8.61	49.41 ± 8.27	NR	NR	NR	NR	Population
Gong Yang et al.	2001	China	7	Case control	1996–1998	143	143	54.00	55.00	NR	NR	NR	NR	Population
Catherine Schairer et al.	2004	USA	8	Case control	1977–1987	185	159	60.90 ± 9.90	67.00 ± 8.30	NR	NR	2.60 ± 2.00	2.60 ± 2.20	Hospital
Juan-Bosco Lopez-Saez et al.	2008	Spain	8	Case control	2004–2006	204	250	50.94	51.05	27.42 ± 6.02	25.80 ± 7.26	NR	NR	Hospital
Insulin														
M. Elisabeth Del Giudice et al.	1998	Canada	7	Nested case control	NR	99	99	43.30 ± 4.90	41.70 ± 5.20	23.80 ± 3.90	24.20 ± 3.80	1.80 ± 1.20	1.50 ± 1.30	Hospital
Helena Jernström et al.	1999	USA	8	Case control	1972–1994	45	393	74.02 ± 8.29	74.32 ± 9.63	25.14 ± 3.40	24.65 ± 4.47	1.73 ± 1.32	1.91 ± 1.58	Population
Paola Muti et al.	2002	Italy	8	Cohort	1987–1992	133	503	51.20 ± 8.41	50.56 ± 8.30	25.12 ± 4.02	25.59 ± 4.53	1.80 ± 1.05	1.99 ± 1.36	Population
Maria Luisa Garmendia et al.	2007	Chile	8	Case control	2000–2005	170	170	56.50 ± 12.30	55.18 ± 10.40	28.59 ± 4.70	29.23 ± 4.61	NR	NR	Hospital
Cun-Zhi Han et al.	2008	China	7	Case control	2001–2005	240	500	45.00	44.00	25.05 ± 3.55	23.36 ± 3.06	NR	NR	Population
Juan-Bosco Lopez-Saez et al.	2008	Spain	8	Case control	2004–2006	204	250	50.94	51.05	27.42 ± 6.02	25.80 ± 7.26	NR	NR	Hospital
Sabina Sieri et al.	2012	Italy	8	Cohort	1987–1992	373	1434	NR	NR	NR	NR	NR	NR	Population
Machiko Minatoya et al.	2013	Japan	8	Case control	2012–2013	63	76	59.85 ± 12.95	56.27 ± 14.78	22.67 ± 3.43	21.16 ± 3.85	1.80 ± 0.53	2.10 ± 0.76	Hospital
Maria Dalamaga et al.	2013	Greece	8	Case control	2003–2010	102	102	61.50 ± 8.20	62.80 ± 8.90	27.70 ± 4.10	25.90 ± 5.40	2.10 ± 0.70	2.30 ± 0.80	Hospital
Hid Felizardo Cordero-Franco et al.	2014	Mexico	8	Case control	2012–2013	124	197	53.20 ± 12.30	55.40 ± 10.50	29.40 ± 5.50	28.80 ± 5.00	NR	NR	Hospital
Machiko Minatoya et al.	2014	Japan	8	Case control	2012–2013	66	66	NR	NR	22.63 ± 3.26	21.60 ± 4.08	2.03 ± 0.74	2.07 ± 0.70	Hospital
Syed Danish Haseen et al.	2015	Pakistan	8	Case control	2010–2014	175	175	46.15 ± 10.58	44.52 ± 10.58	21.61 ± 4.10	21.60 ± 3.70	NR	NR	Hospital
Geoffrey C. Kabat et al.	2018	USA	8	Nested case control	1993–1998	1,185	19632	63.90 ± 7.10	64.00 ± 7.30	30.40 ± 6.10	29.60 ± 6.20	2.70 ± 1.80	2.70 ± 1.70	Population
Poonam Kachhawa et al.	2018	India	7	Case control	NR	253	258	50.50 ± 10.80	49.50 ± 11.00	23.50 ± 2.39	23.10 ± 2.36	NR	NR	NR

BMI, body mass index; NR, not reported.

*Population-based control means that cases in the control group of the study are from a community, a city screening center, or a permanent residence. Hospital-based control means that cases in the control group of the study are from a hospital.

**Table 2:** The levels of serum insulin or C-peptide in each primary study.

Author	Year	Premenopausal		Postmenopausal		Cases			Control			Unit	Method
		Cases	Control	Cases	Control	Mean	SD	N	Mean	SD	N		
C-peptide													
Helena Jernström et al.	1999	0	0	45	391	1.97	1.44	45	1.89	0.92	391	ng/ml	RIA
Paolo Toniolo et al.	2000	172	486	115	220	3.58	0.15	287	3.53	0.09	706	ng/ml	RIA
Gong Yang et al.	2001	45	45	98	98	1.40	1.33	143	1.11	0.88	143	ng/ml	ELISA
Catherine Schairer et al.	2004	0	0	185	159	1.70	1.04	185	1.60	0.64	159	ng/ml	RIA
Juan-Bosco Lopez-Saez et al.	2008	108	125	96	125	3.04	1.30	204	1.96	1.16	250	ng/ml	ELISA
Insulin													
M. Elisabeth Del Giudice et al.	1998	99	99	0	0	6.69	0.21	99	6.20	0.19	99	μIU/ml	RIA
Helena Jernström et al.	1999	0	0	45	390	3.81	3.95	45	3.23	2.56	390	pmol/L	RIA
Paola Muti et al.	2002	69	265	64	238	10.14	6.54	133	9.95	6.19	503	μIU/ml	RIA
Maria Luisa Garmendia et al.	2007	48	54	122	116	14.18	8.95	170	14.45	9.61	170	μIU/ml	ELISA
Cun-Zhi Han et al.	2008	NR	NR	NR	NR	13.30	10.56	240	6.34	4.48	500	μIU/ml	ELISA
Juan-Bosco Lopez-Saez et al.	2008	108	125	96	125	70.02	5.78	204	80.93	10.36	250	mmol/L	ELISA
Sabina Sieri et al.	2012	NR	NR	NR	NR	6.52	4.90	373	6.18	6.90	1434	μIU/ml	CLEIA
Machiko Minatoya et al.	2013	22	31	41	45	7.50	7.70	63	7.50	6.60	76	μIU/ml	CLEIA
Maria Dalamaga et al.	2013	0	0	102	102	11.90	11.30	102	9.40	6.50	102	μIU/ml	NR
Hid Felizardo Cordero-Franco et al.	2014	48	70	76	127	11.60	8.20	124	14.20	8.60	197	μIU/ml	ELISA
Machiko Minatoya et al.	2014	22	22	44	44	7.63	7.54	66	7.47	6.21	66	μIU/ml	CLEIA
Syed Danish Haseen et al.	2015	69	83	106	92	19.76	9.66	175	15.41	8.07	175	μIU/ml	ELISA
Geoffrey C. Kabat et al.	2018	0	0	1,185	19,632	74.80	83.60	1,185	67.80	103.60	19632	mg/dl	ELISA
Poonam Kachhawa et al.	2018	110	117	143	141	14.08	6.29	253	12.56	5.36	258	μIU/ml	ELISA

BMI, body mass index; NR, no report; RIA, radioimmunoassay; ELISA, enzyme-linked immunosorbent assay; CLEIA, chemiluminescent enzyme immunoassay; ELISA, SD, standard deviation

### Overall Analysis and Subgroup Analysis

**Figure 2:**
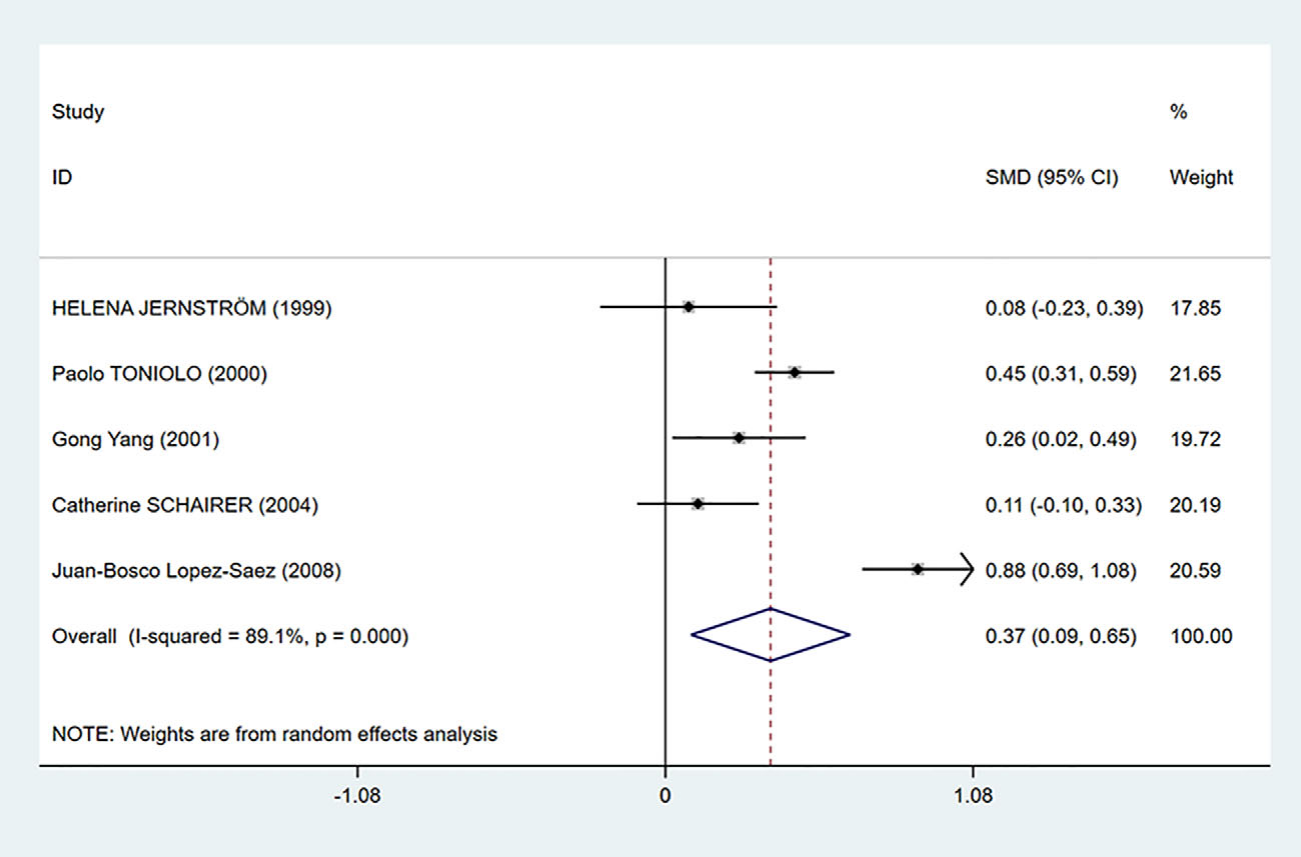
Overall forest plot of meta-analysis on the association between C-peptide and breast cancer risk. The size of the square box is proportional to the weight that each study contributes in the meta-analysis. The overall estimate and CI are marked by a diamond. Summary estimates were analyzed using a random-effects model. Zero is not included in this confidence interval, and diamond is on the right of zero, which indicates that C-peptide levels were positively associated with breast cancer risk.

As shown in **Figure 2**, C-peptide levels were significantly positively related to breast cancer (SMD = 0.37, 95% CI: 0.09–0.65). However, there was considerable heterogeneity among the studies (I^2^ = 89.1%, *P* < 0.001). Therefore, we performed subgroup analysis to explore the source of heterogeneity by country, test methods, control source, and menopausal status in our dataset.

**Figure 3:**
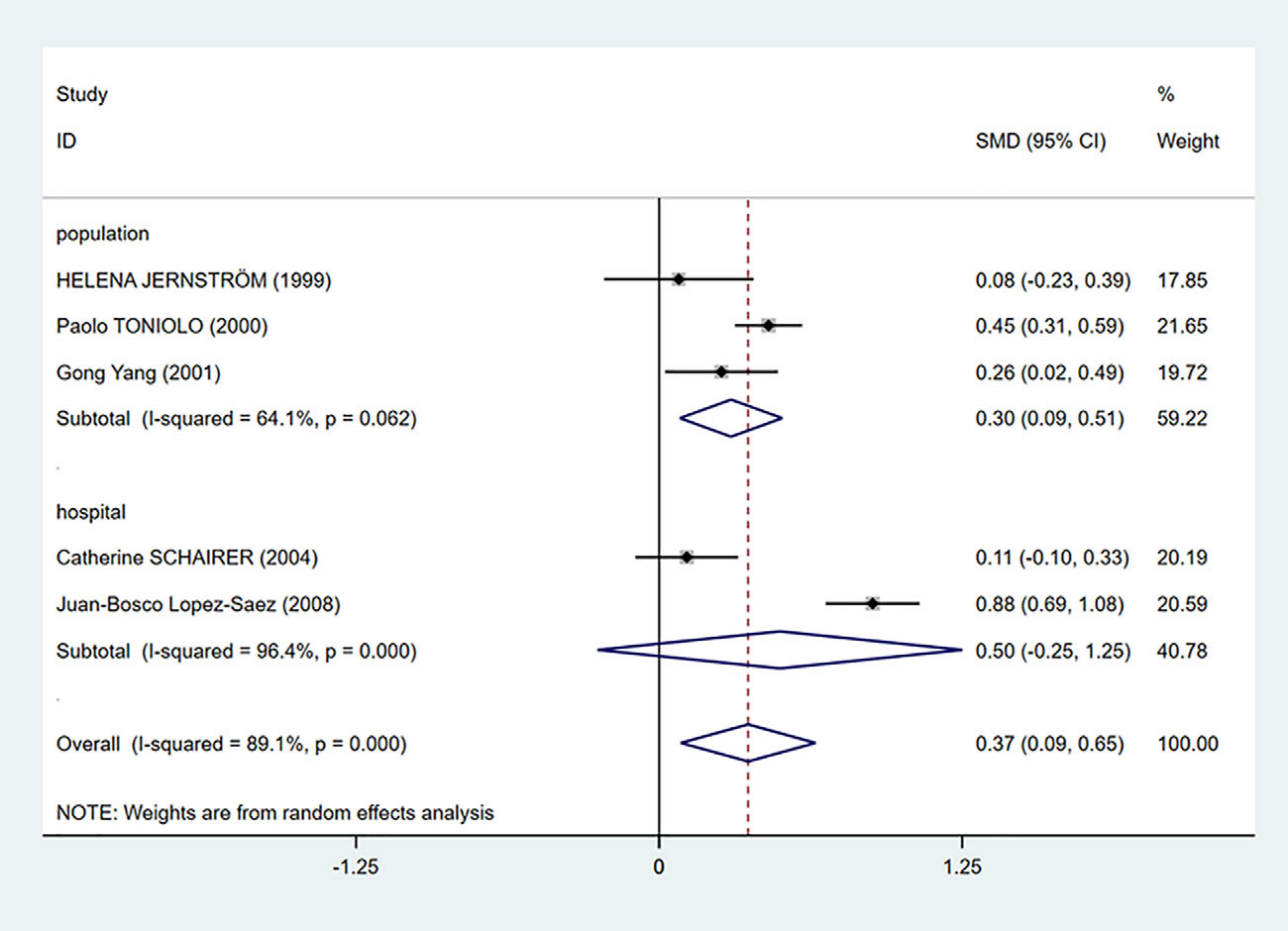
Subgroup analysis of the association between C-peptide and breast cancer in different control sources. The size of the square box is proportional to the weight that each study contributes in the meta-analysis. The overall estimate and CI are marked by a diamond. Summary estimates were analyzed using a random-effects model. C-peptide levels are positively associated with breast cancer risk in population-based control group. No association is presented in hospital-based control group.

We performed a subgroup analysis according to the source of cases in the control groups. Population-based control means that cases in the control group of the study are from communities, city screening centers, or permanent residences. Hospital-based control means that cases in control group of the study are from a hospital. As we can see in **Figure 3**, the mean C-peptide levels of breast cancer patients were significantly higher in the population-based control group (SMD = 0.30, 95% CI: 0.09–0.51, *P* = 0.006), which meant that c-peptide levels were positively correlated with breast cancer in this group. In addition, the heterogeneity of population-based control group decreased (I^2^ = 64.1%, *P* = 0.062).

**Figure 4:**
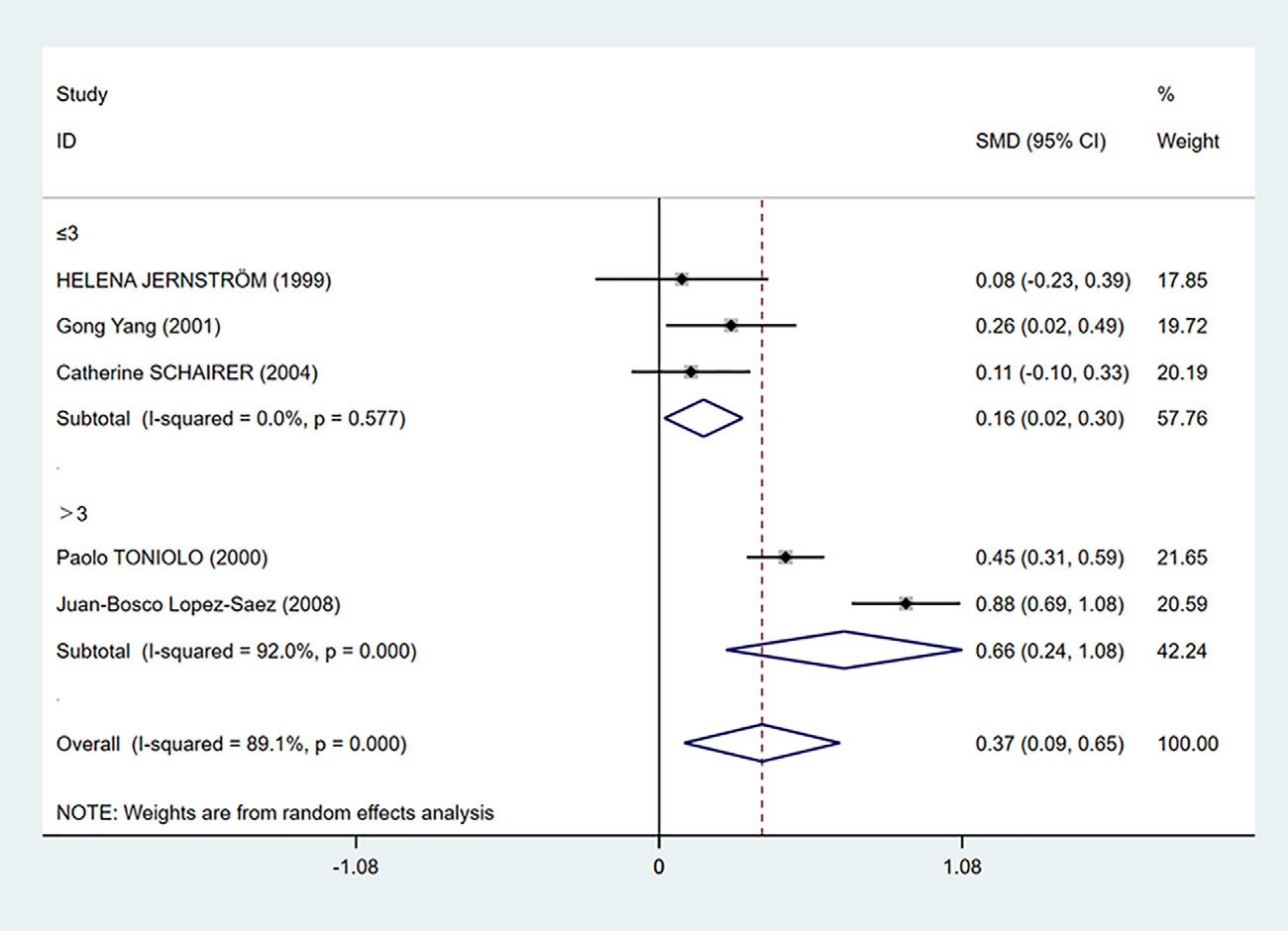
Subgroup analysis of the association between C-peptide levels and breast cancer in C-peptide ≤3 or >3 ng/ml. The size of the square box is proportional to the weight that each study contributes in the meta-analysis. The overall estimate and CI are marked by a diamond. Summary estimates were analyzed using a random-effects model. C-peptide levels are positively associated with breast cancer risk in both ≤3 and >3 ng/ml groups.

To further investigate the source of heterogeneity, we separated five studies into two groups according to the mean C-peptide level in the case group: ≤3 ng/ml and >3 ng/ml group. We found that C-peptide and breast cancer had positive correlations in both groups. The heterogeneity in the ≤3 ng/ml group was decreased dramatically (I^2^ = 0.0%, *P* = 0.577), while the heterogeneity in the >3 ng/ml group increased (**Figure 4**).

**Figure 5:**
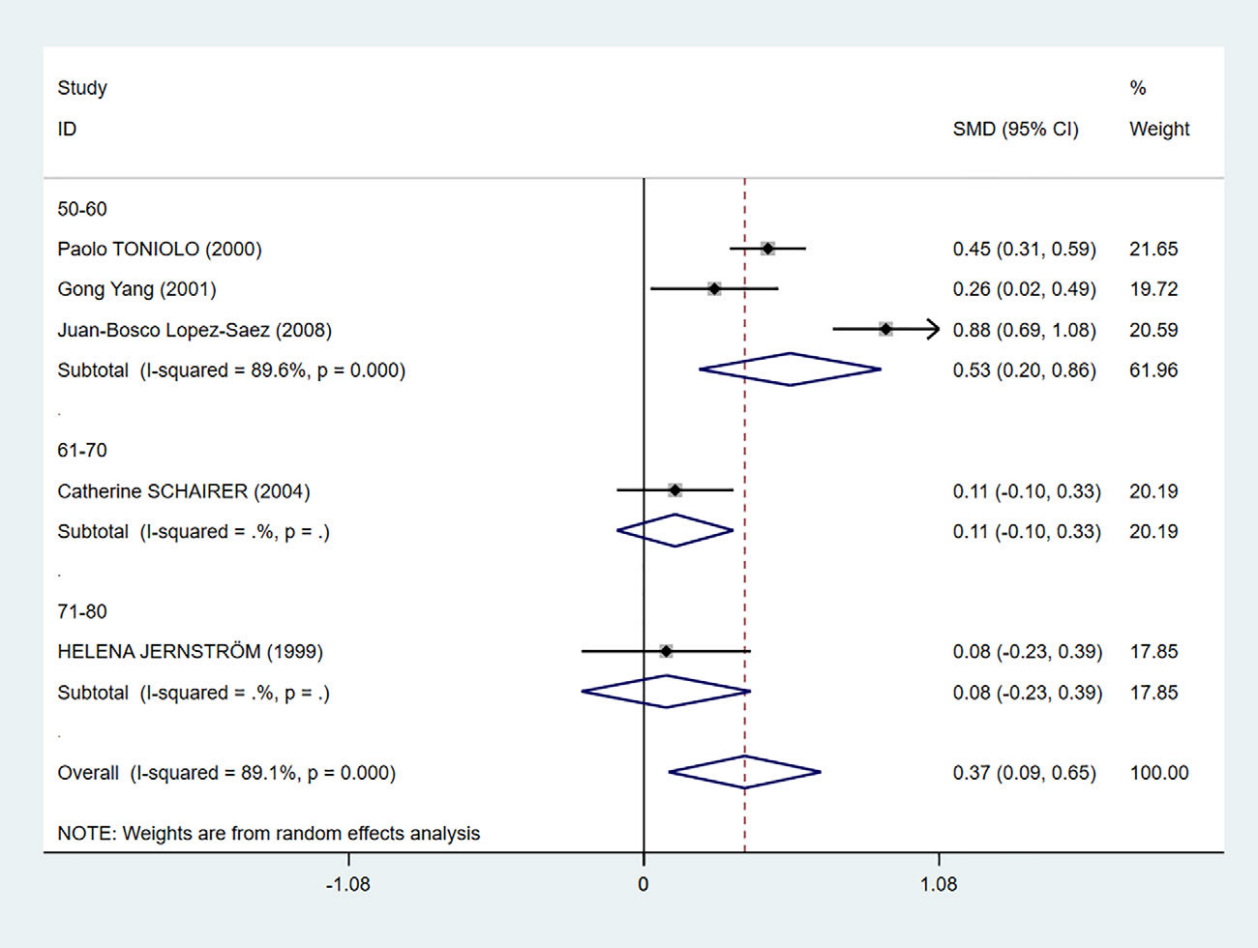
Subgroup analysis of the association between C-peptide and breast cancer in different ages. The size of the square box is proportional to the weight that each study contributes in the meta-analysis. The overall estimate and CI are marked by a diamond. Summary estimates were analyzed using a random-effects model. C-peptide levels are positively associated with breast cancer risk in the age 50–60 group.

We also performed a subgroup analysis based on age (**Figure 5**). Even though there was still heterogeneity, it was obvious that C-peptide level was positively related to breast cancer in women between the ages of 50 and 60 (I^2^ = 89.6%, *P* = 0.000).

In addition, we conducted subgroup analysis according to country, measuring method, and menopausal status, but no source of heterogeneity was found (**Table 3**).

**Table 3:** The pooled and subgroup results of the serum C-peptide levels in breast cancer patients compared with the control groups.

	Number	SMD	95%CI	P	I^2^ (100%)	Model
Overall	5	0.37	0.09–0.65	0.000	89.1	Random effects
Control source						
Population	3	0.30	0.09–0.51	0.062	64.1	Random effects
Hospital	2	0.50	−0.25-1.25	0.000	96.4	Random effects
C-peptide level (ng/ml)						
≤3	3	0.16	0.02–0.30	0.577	0.0	Random effects
>3	2	0.66	0.24–1.08	0.000	92.0	Random effects
Country						
USA	3	0.24	−0.03–0.50	0.009	78.7	Random effects
China	1	0.26	0.02–0.49	–	–	Random effects
Spain	1	0.88	0.69–1.08	–	–	Random effects
Method						
RIA	3	0.24	−0.03–0.50	0.009	78.7	Random effects
ELISA	2	0.57	−0.04–0.65	0.000	93.9	Random effects
Menopausal						
Premenstrual	2	1.18	−6.07–8.43	0.000	99.9	Random effects
Postmenstrual	4	0.76	−0.11–1.63	0.000	97.7	Random effects
Both	1	0.26	0.02–0.49	–	–	Random effects
Age						
50–60	3	0.53	0.20–0.86	0.000	89.6	Random effects
61–70	1	0.11	−0.10–0.33	–	–	Random effects
71–80	1	0.08	−0.23–0.39	–	–	Random effects

SMD, standardized mean difference; CI, confidence interval; ELISA, enzyme-linked immunosorbent assay; RIA, radioimmunoassay.

**Figure 6:**
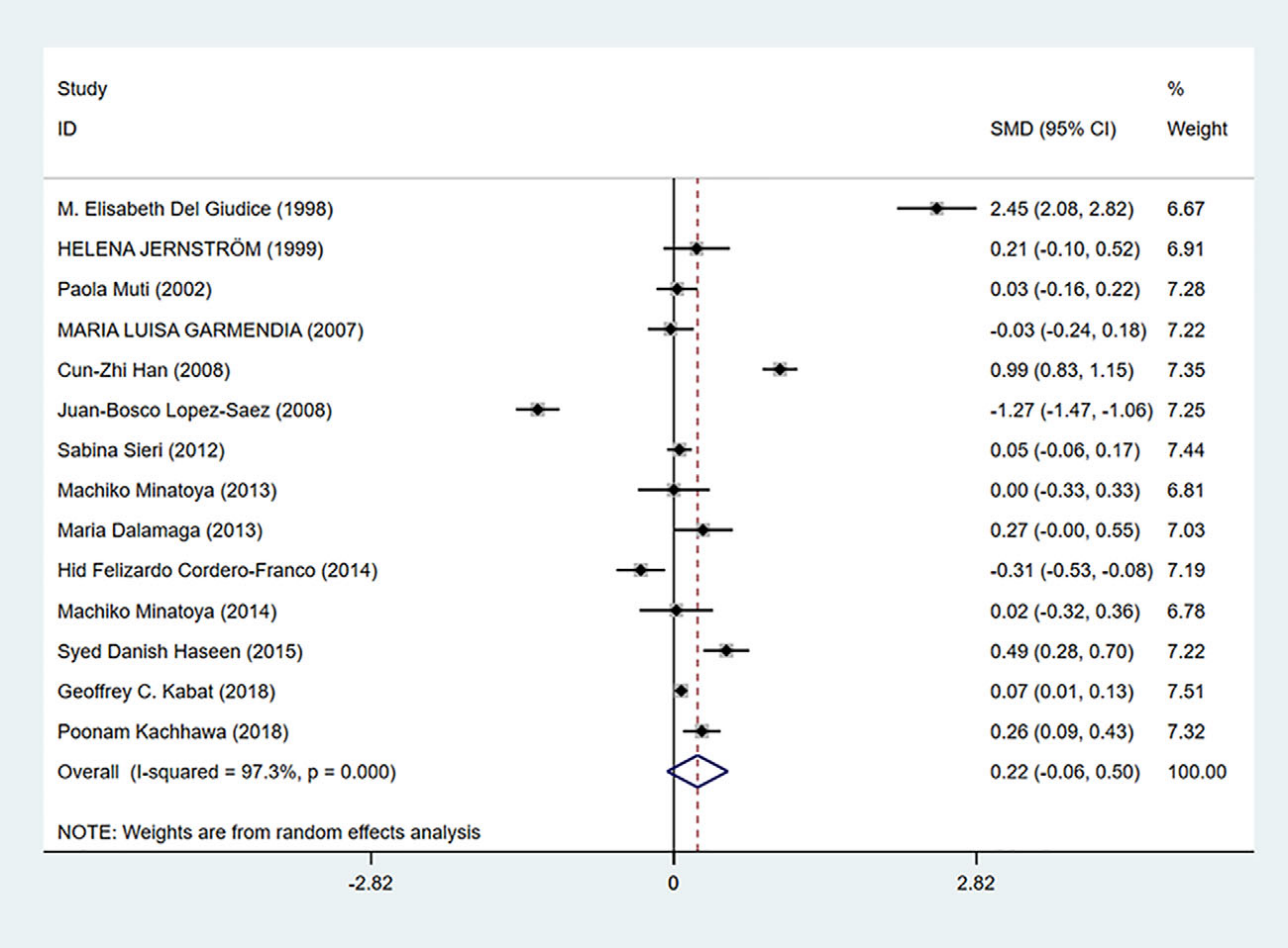
Overall forest plot of meta-analysis on the association between insulin and breast cancer risk. The size of the square box is proportional to the weight that each study contributes in the meta-analysis. The overall estimate and CI are marked by a diamond. Summary estimates were analyzed using a random-effects model. Zero is included in this confidence interval, which indicates that insulin levels had no association with breast cancer risk.

As shown in **Figure 6**, insulin levels were irrelevant to breast cancer (SMD = 0.22, 95% CI: −0.06–0.50) and considerable heterogeneity exists among studies (I^2^ = 97.3%, *P* = 0.000).

### Publication Bias and Sensitivity Analysis

**Figure 7:**
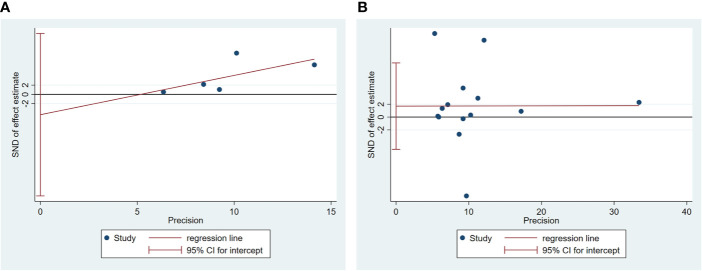
Egger’s linear regression for publication bias about association between C-peptide **(A)** or insulin **(B)** and breast cancer. Each circle represents a separate study pertaining to the indicated association. The circles imply that an asymmetrical distribution is not present, suggesting that no publication biases were present.

**Figure 8:**
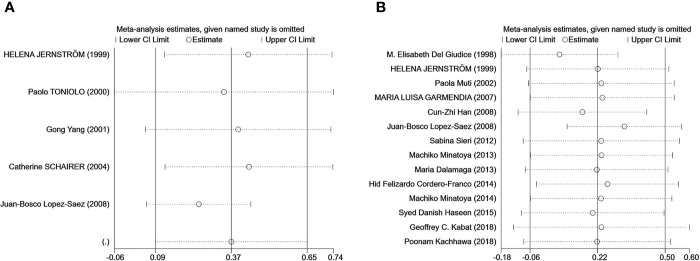
The result of the sensitivity analysis on the association between C-peptide **(A)** or insulin **(B)** and breast cancer. Sensitivity analyses for the influence of individual studies on the summary SMD. The vertical axis indicates the overall SMD, and the two vertical axes indicate its 95% CI. Every hollow round indicates the pooled SMD when the left study is omitted in this meta-analysis. The two ends of every broken line represent the respective 95% CI.

Egger’s test was used to measure publication bias (*P* = 0.48 for c-peptide and *P* = 0.59 for insulin). We found no publication bias among the articles included in this meta-analysis (**Figure 7**). The sensitivity analysis demonstrated the stability of our meta-analysis (**Figure 8**).

## Discussion

The overall conclusion of this study was consistent with those of previous articles indicating no relationship between serum insulin and breast cancer. Autier et al. (16) found that serum insulin levels showed a non-significant increase in the risk of breast cancer in a previous meta-analysis. The meta-analysis of Hernandez et al. (4) also found that insulin had little effect on the incidence of breast cancer. However, several epidemiologic studies (34–36), but not all (10, 37), showed that high levels of insulin cycling were related to increased breast cancer incidence in premenopausal and postmenopausal women. Some laboratory studies showed that insulin, like its associated growth factors, may have mitotic effects on both normal and neoplastic breast epithelial cells (38, 39). This could be caused by the characteristics of insulin that its half time in serum is short. Metabolism of insulin in blood is complex and more study is needed in order to clarify the relationship among insulin, metabolism and cancer.

As shown in the results, we analyzed the correlation between C-peptide and breast cancer. Although this study drew the same conclusion as previous studies reporting that C-peptide is of great importance in breast cancer progression (4, 20), there was considerable heterogeneity among studies (I^2^ = 89.1%). We found no publication bias among the included articles, and sensitivity analysis showed the superior reliability of this meta-analysis, which demonstrated that the conclusion is reliable.

To further identify the source of the heterogeneity, we did subgroup analysis using several factors, including country, measuring method, control source, age, and menopausal status, which may increase variability of results. In the subgroup analysis by control source, we found that the mean C-peptide levels of breast cancer patients were higher in the population-based control subgroup and the heterogeneity decreased significantly, which means that serum C-peptide level was positively related to breast cancer in this group. Nevertheless, the hospital-based control subgroup showed no relationship between C-peptide and breast cancer level. This situation may be explained by the fact that control patients from hospital suffered from diseases even though they were not diagnosed, which amplified the heterogeneity of the hospital control source group. Although we excluded studies that included patients with a combination of other diseases in the *Methods* section, we cannot guarantee the quality of each patient in each article.

Furthermore, we performed a subgroup analysis in which studies were divided into two groups according to the C-peptide levels of breast cancer patients. The results showed that even though the heterogeneity in the ≤3 ng/ml group decreased dramatically, C-peptide level in the >3 ng/ml group was higher than that in the ≤3 ng/ml group. It was reported that insulin resistance, characterized by elevated C-peptide levels, was associated with central obesity and several other diseases, including cancer (38, 40). This is consistent with the result in this meta-analysis to the fact that the higher C-peptide level, the greater the risk of breast cancer. However, we did not find the source of heterogeneity when performing subgroup according to country, test method or menopausal status.

In subgroup analysis according to age, C-peptide level was positively related to breast cancer in women between the ages of 50 and 60 (**Figure 5**). It is reported that 67.8% breast cancer women are at the age of 41–60 (41), which may be for the reason that women are just entering menopause, and metabolism is in a state of disorder.

However, our results were inconsistent with those of a previous meta-analysis reporting that C-peptide did not play a role in breast cancer progression. Hernandez et al. (42) found that elevated fasting insulin or C-peptide levels were not related to breast cancer in women. Autier in 2013 reported that they did not find any results to locate a positive relationship between insulin or C-peptide levels and breast cancer incidence (16). They calculated summary relative risks and 95% CI applied to the relative risk associated with the highest *versus* lowest quantile of serum concentrations. However, we employed the sample size, the mean, and SD to calculate SMDs and 95% CIs for each study, which is the better way to do meta-analysis of continuous variable (43). In addition, some studies reported that C-peptide correlated positively with breast cancer risk, but only among postmenopausal women (44). In our analysis, two articles reported data about postmenopausal women, so there were more cases in postmenopausal women than that in premenopausal women, which may explain our conclusion. In addition, insulin and C-peptide are synthesized in islets by enzymatic hydrolysis of proinsulin and released into the blood circulation in equal moles. C-peptide has a longer half-life in plasma than insulin; therefore, it can serve as a marker to more accurately reflect the relationship between metabolism and breast cancer (45).

There are some limitations in our meta-analysis. First, in terms of medical ethics, some studies have relatively limited sample sizes. Second, because of the limited number of included studies, we were unable to conduct an adequate combinatorial analysis during the subgroup analysis nor were we able to analyze many other parameters that might reflect breast cancer (*e.g.*, breast cancer type, breast cancer stage, and BMI, *etc*.). Third, unmeasured and residual confounding may result in confusion in relation to the conclusion. Finally, the analysis was also limited by the quality of the individual studies, and many other important factors affecting breast cancer incidence could not be analyzed. Given the limitations of our meta-analysis, further large-scale studies and sufficient samples are needed in order to present a convincing link between insulin and C-peptide and breast cancer risk.

## Conclusion

The current meta-analysis suggests that C-peptide levels were positively related to breast cancer risk in women, and no relationship between insulin and breast cancer was found.

## Data Availability Statement

All datasets presented in this study are included in the article/supplementary material.

## Author Contributions

ML and LS designed the program, searched and reviewed the studies, and were in charge of the manuscript. ML, JY, and DZ assessed the studies, extracted the data, and wrote part of the manuscript. LS, CZ, and YLiu extracted the data and wrote part of the manuscript. QL, HW, KS, YLi, ZM, and DL reviewed and edited the manuscript. JD directed the project, contributed to the discussion, reviewed, and edited the manuscript. All authors contributed to the article and approved the submitted version.

## Funding

This study was supported by grants (to JD) from the National Natural Science Foundation of China (No. 31872791) and Natural Science Foundation of Shandong Province of China (No. ZR2019MC046).

## Conflict of Interest

The authors declare that the research was conducted in the absence of any commercial or financial relationships that could be construed as a potential conflict of interest.

## Abbreviations

SMDs, standardized mean differences; CIs, confidence intervals; BMI, body mass index; NOS, Newcastle–Ottawa Scale; SD, standard deviation; ORs, odds ratios.
